# Alterations in locomotor activity of feeding zebrafish larvae as a consequence of exposure to different environmental factors

**DOI:** 10.1007/s11356-016-6704-3

**Published:** 2016-04-27

**Authors:** Renate Kopp, Juliette Legler, Jessica Legradi

**Affiliations:** Institute for Environmental Studies (IVM), VU University Amsterdam, De Boelelaan 1085, 1081 HV Amsterdam, The Netherlands

**Keywords:** Zebrafish, Locomotor activity, Light, Sound/vibrations, Diet, Environmental stimuli

## Abstract

Behavioral studies are important tools for understanding the development and pathology of neurological diseases. Zebrafish are an emerging alternative model in behavioral and neurological studies as the behavioral repertoire of zebrafish (*Danio rerio*) is similar to humans, and nervous system structures and functions are highly conserved. In this study, we investigated alterations in day/night locomotor activity of free swimming, feeding wild-type zebrafish larvae (8–15dpf) due to changes in the rhythm of light/dark cycles or caloric content of food. We furthermore exposed zebrafish larvae to continuous stress by applying alternated minor vibrations. Under altered rhythms of light/dark cycle’s zebrafish larvae still expressed a distinct light/dark activity pattern but the total activity was reduced compared to control animals. When the larvae were exposed to continuous light, they still had coordinated resting cycles but maximal activity and excitation rates after feeding were increased, indicating that food became the new zeitgeber. Feeding food of high caloric content induced continuously high activity levels during light cycles and significantly elevated activity levels during the dark. Exposure to continuous vibrations lowered total activity levels. We showed previously that changes in environmental factors like light/dark cycles or changes in caloric content of food can affect adipogenesis, lipid composition, and circadian rhythm of free swimming, feeding larvae but this is the first time showing how theses factor alter behavior.

## Introduction

The zebrafish (*Danio rerio*), a well-established model species in developmental biology, represents an emerging alternative model in behavioral and neurological studies. Their behavioral repertoire is similar to rodents and humans (Kalueff et al. 2013), and nervous system structures and functions are highly conserved (Ingebretson and Masino 2013). Within 120 h of embryonic development, zebrafish fully develop a complex nervous system (Burgess and Granato 2007) (Blader and Strahle 2000). After 1 week of development, zebrafish larvae already display behavioral patterns as complex as adults. Zebrafish larvae display a defined locomotor repertoire as they swim, turn and capture pray (Budick and O’Malley 2000). The behavior of zebrafish can easily be monitored by automated video tracking systems, and due the larvae’s small size, these experiments can be performed in multiwell plates, petridishes, or small tanks during the development up to adulthood. Zebrafish behavioral tests are increasingly used for neurotoxicity studies (Ton et al. 2006) (Legradi et al. 2014), as well as for investigating neurological diseases like Alzheimer’s disease (Newman et al. 2014) or dementia (Willlemsen et al. 2010).

Zebrafish behavior, similar to humans, can be influenced by environmental factors like light and temperature changes and external stimuli like chemicals or sound/vibrations. Activity and resting periods are determined by circadian rhythms, thus by daily rhythms of light/dark cycles (LD). Like humans, zebrafish show a diurnal activity pattern and rest during the dark period. From around 20 h post fertilization on zebrafish react to light (Kazimi and Cahill 1999). After the first day of development, they respond with heavy tail twitches to a short light flash (Kokel et al. 2010), and with 5 days post-fertilization (dpf), a distinct day night activity pattern is expressed (Woods et al. 2014).

Zebrafish are also highly sensitive to vibrations and react with a strong escape response. For vibration detection, zebrafish develop two mechanosensory systems: the inner ear and the lateral line. Zebrafish have Weberian ossicles, specialized bones that mechanically connect the swim bladder to the hearing organs and from 5 dpf on, when the otoliths are calcified and the swim bladder inflated, zebrafish larvae respond with startling to acoustic signals (Zeddies and Fay 2005). Vibrations can be seen as one of the strongest stress stimulus and zebrafish regulate adaptive stress responses by the hypothalamus-pituitary-interrenal axis in a similar way as humans do (Cachat et al. 2010).

Generally, zebrafish react highly sensitively to changes in the environment. There are many studies investigating the effect of environmental factors on behavior in larvae up to 6 dpf (e.g., short light/dark transitions). It is also known that changes in feeding and light/dark cycles can alter swimming behavior. It has been shown that zebrafish larvae kept in darkness display a shift in activity pattern, and this effect is linked to changes in circadian clock genes (per1) (Huang et al. 2015). Also the biphasic effect of food deprivation on activity patterns leading first to a higher activity followed by a reduced activity was shown (Novak et al. 2005). But it is not known how changes in environmental factors, like caloric food content, shifts in day/night cycles, and exposure to continue stress like vibrations stimuli affect normal baseline behavior of older larvae. In a previous study, we could already demonstrate that alterations in day/night cycles and food content clearly affect adipogenesis, lipid composition, and circadian rhythms of zebrafish larvae (Kopp et al. 2015) but we did not investigated the effect on behavioral activity. To gain more insights about changes of activity patterns in response to environmental factors, we performed the following study. We investigated alterations in locomotor activity of developing Tubingen Longfin (TL) wild-type zebrafish to changes in rhythm of light/dark cycles or caloric content of food. We furthermore exposed zebrafish larvae to continuous stress as sound/vibrations during a period of 7 days. By comparing total activity, day/night activity, and excitation rate in response to different environmental factors, we were able to clearly show that changes in light/dark cycles, food, and exposure to continuous vibrations significantly affect baseline locomotor behavior.

## Material and methods

### Ethics statement

This study was carried out in strict accordance with the recommendations in care and use of laboratory animals of the directive of the Dutch Parliament. The protocol was approved by the Committee on the Ethics of Animal Experiments of the VU University of Amsterdam (Permit Number: DEC IVM 13–01). All efforts were made to minimize suffering.

### Experimental design

Experiments were performed with TL wildtype zebrafish (*Danio rerio*) raised in tanks of about 30 individuals. Soon after spawning, eggs out of several tanks were blended and transferred to embryonic medium (5 mM NaCl, 0.17 mM KCl, 0.33 mM CaCl2, and 0.33 mM MgSO4) and embryos and larvae raised in temperature (26 °C) and light (14/10 light/dark) controlled incubators. As light source a BeamsWork Power LED 200 (10.000 K daylight, 200 lumen) was used.

**Fig. 1:**
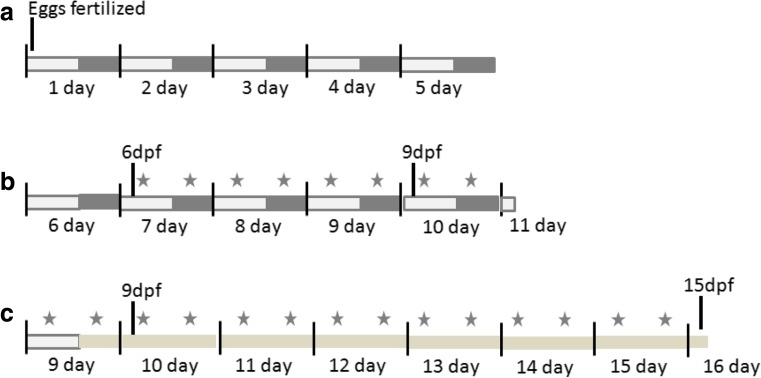
Overview of the experimental design. Zebrafish were raised with constant light/dark cycles till day 9. From 6dpf on larvae were feed twice a day, one time during the light period, and one time during the dark period. Differences in light condition or vibration started at day 9, and locomotion was monitored till 15dpf

From 6 days post-fertilization (dpf) on, constant volumes of *Tetrahymena* suspension plus 6 mg powdered baby food (Sera micron) were added twice a day. For hypercaloric diet (HCD), one feeding was replaced by boiled chicken egg yolk which was suspended in embryonic medium, and 4 ml of the suspension was fed. Larvae were fed 1 hour after lights-on (zeitgeber time ZT + 1) and 2 hours after lights-off (ZT + 16). These two time points were selected to ensure comparable feeding conditions also when altered light/dark protocols (jet lag, alternating shortened, and prolonged days according to Kopp et al., 2015 were applied). An overview of the experimental scheme is shown in Fig. 1c. The monitoring always started under standard conditions (8 dpf) to normalize experiments and to check for normal behavior of the larvae before treatment. At day 9, different treatment conditions were started: jet lag, continuous light (LL), and sound exposure.

**Fig. 2:**
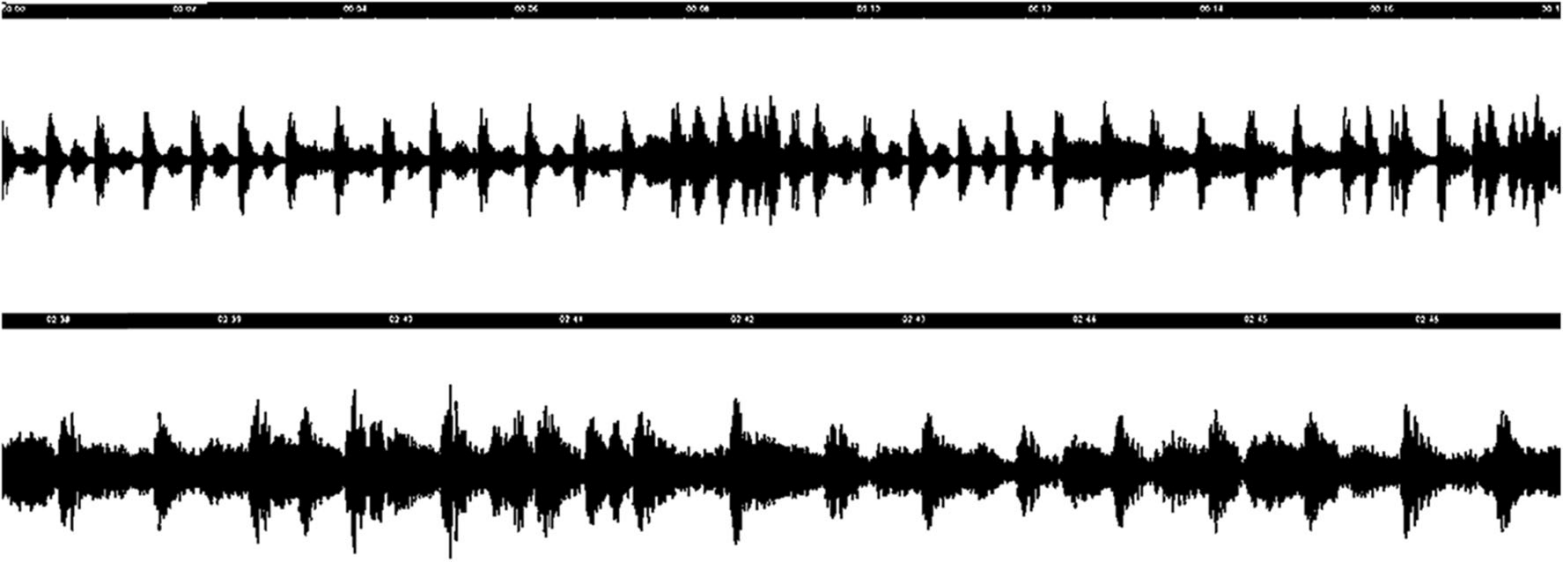
Sound waves of the vibration experiment

For sound experiments, the vibration module of the ZebraBox (ViewPoint) system was used, which transfers loudspeaker vibrations directly to a multi well plate. Zebrafish larvae were exposed to an endless repeat of alternating sound/vibrations. The sound was selected to ensure an irregular vibration pattern with only moderate changes in amplitude (Fig. 2). Thus, a habituation effect as well as occasional overexposure could be minimized. Sound intensity was adjusted, avoiding visual vibrations on the larvae, multiwall plate, or medium.

### Behavioral analysis

Each well of a transparent 6-well plate was filled with embryonic medium and 10 zebrafish larvae. Sample size was selected to minimize number of animals but to still be able to detect significant differences and assure animal welfare (group behavior). The 6-well plate was added to a temperature controlled automated observation system (ViewPoint ZebraBox). A controllable white light source suitable for circadian cycle simulation and an infrared high sensitivity camera with high image definition allowed for monitoring locomotor activity over a period of 7 days. Twice a day, the larvae were fed, and the medium was refreshed once a day right before feeding. To avoid interfering signal by the medium exchange, this was done outside the observation system.

Per well activity curves were established by monitoring the time all larvae in a well were active (movement threshold 7 cm/s) within 10 min. Thus, data of each experimental group consists of 6 activity curves produced by 10 larvae each. For each activity curve, maximal activity values of the ninth observation day were set as 100 % to exclude individual variation. Total activity was determined by calculating the area below the activity curves. After feeding and lights-on, excitation (maximal response to a stimulus in % based on the whole experimental period) and latency (time in minutes to reach the maximum activity after a stimulus) were read out of the curve for each stimulus event. To improve comparability between experimental groups, excitation values were divided by associated latency values. The resulting excitation rate was calculated for each stimulus event, and mean values over the total experimental period were compared with control values as an indicator of hypo or hyperactivity due to stimulus.

### Statistics

Total activity and excitation rate values of all experimental groups were compared to values of control conditions. Statistical differences were calculated by applying one-way ANOVA on ranks, and an adequate post hoc test and significance was accepted when *p* < 0.05. To identify significant difference between treatment groups and over time, a repeated measures ANOVA considering time and groups was applied. Significance was accepted when the adjusted *p* < 0.05 (Greenhouse-Geisser, Huynh-Feldt, and Lower bound corrections). Statistical analyses were performed using Excel software (2007) and Matlab (version R2014a, The MathWorks, Natrick, MA, USA).

## Results

### Control conditions

**Fig. 3:**
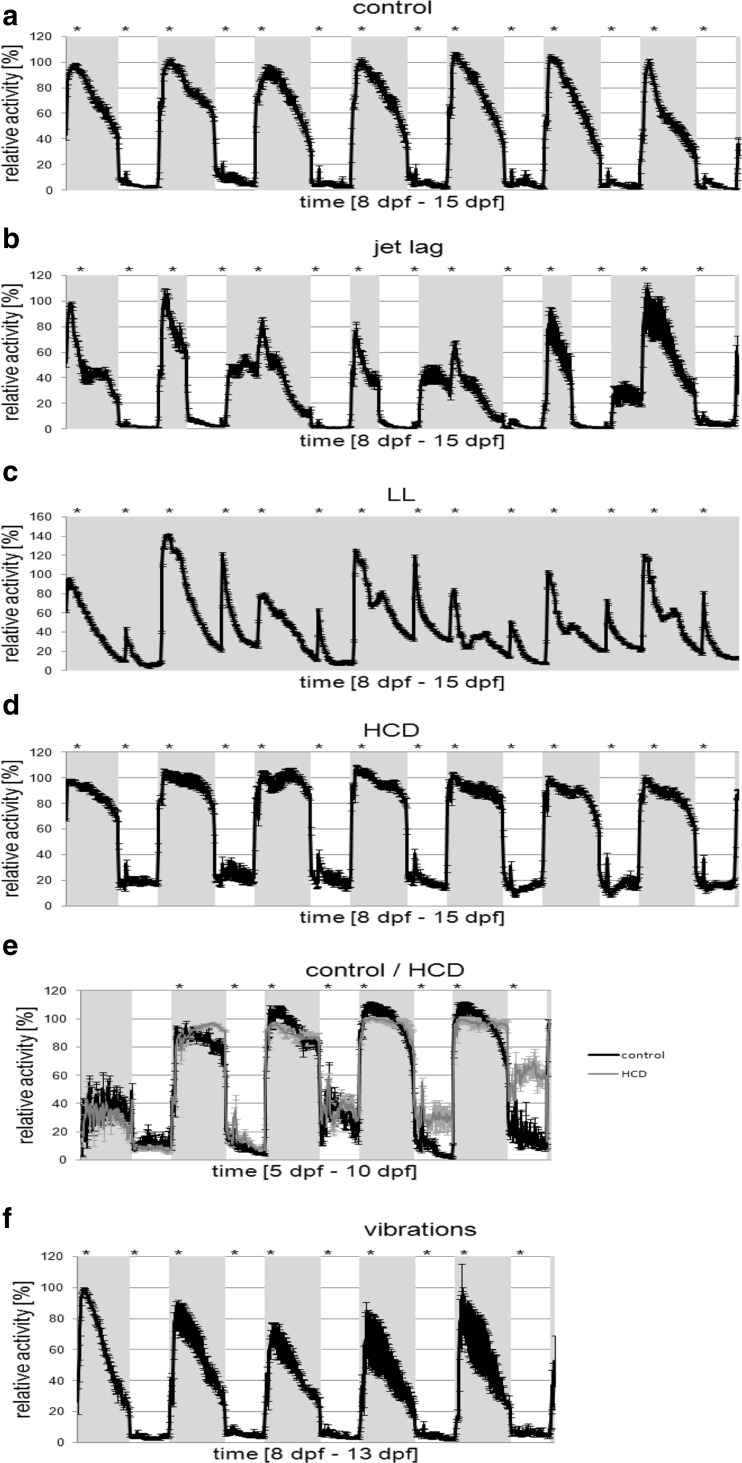
Activity patterns (relative activity in %) throughout different experimental conditions. Light-on is marked with gray shading. Feeding time is marked with *. The scale of the y- and x-axis differs between graphs

**Fig. 4:**
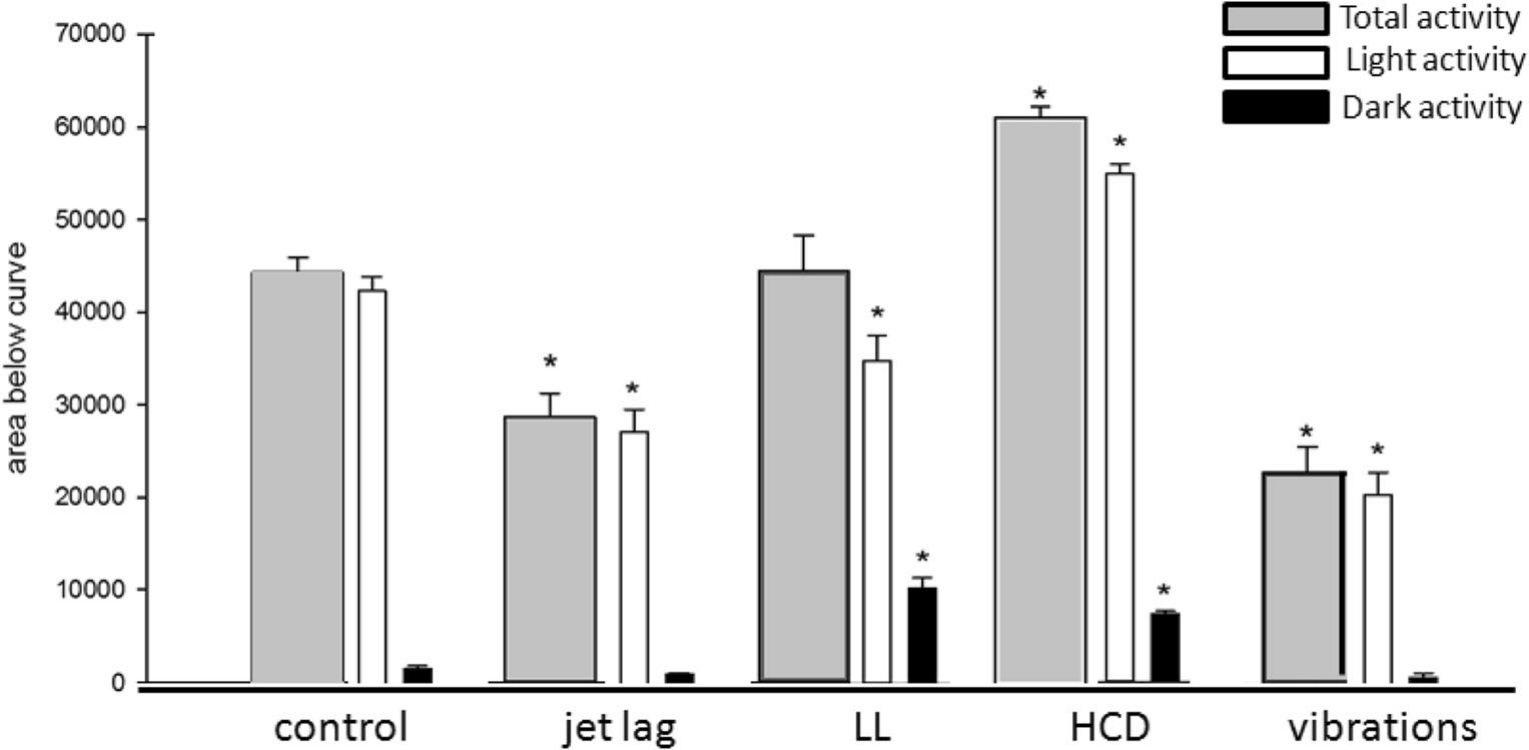
Total activity calculated as area under the activity curve covering the whole experimental period and subdivided into lights-on and lights-off periods. For the LL group, lights-off periods were calculated for the corresponding time window of the control group

**Fig. 5:**
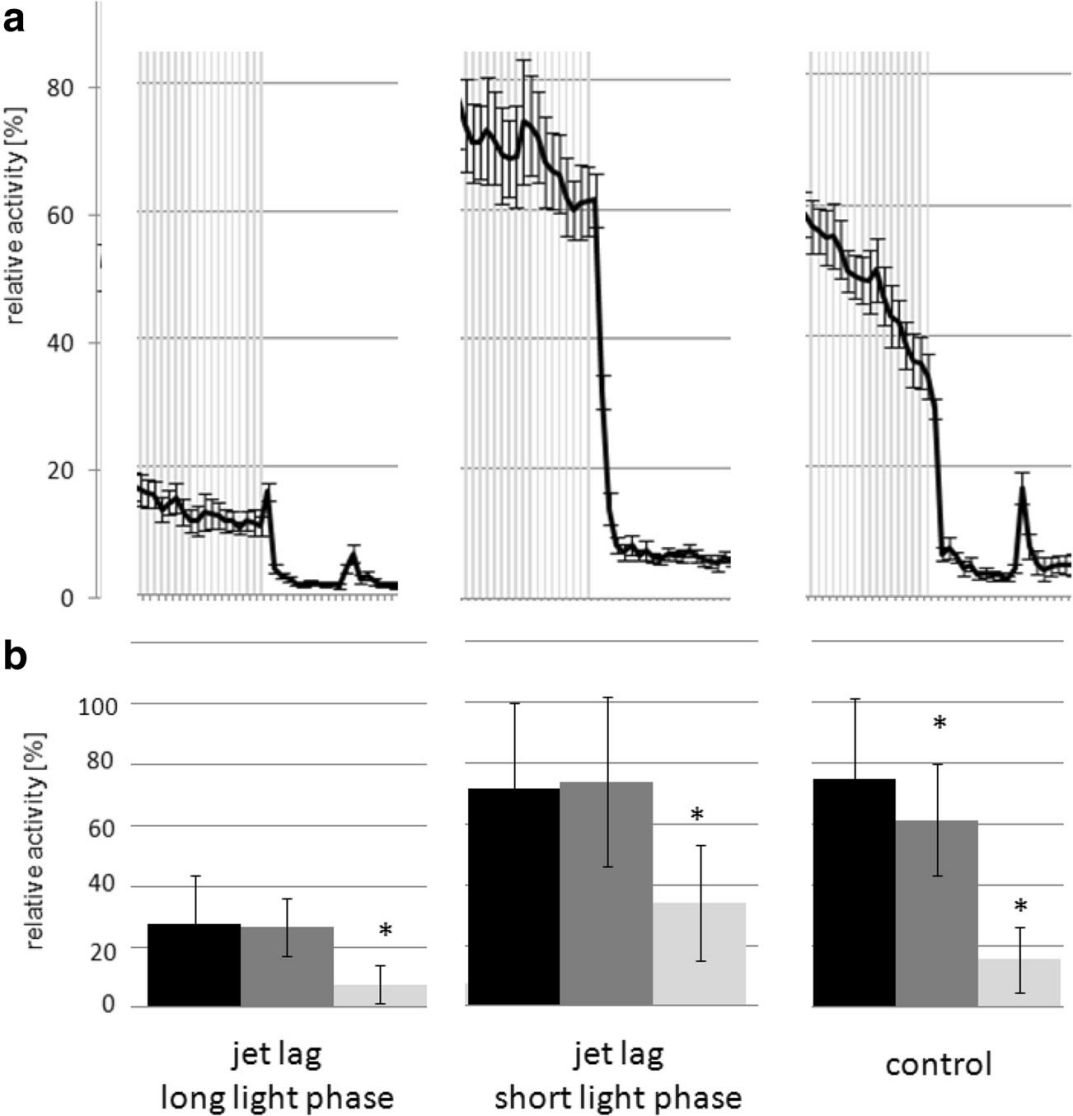
**a** Detailed view of the response to lights off for jet lag (long and short light phase) and control. Activity patterns (relative activity in %) throughout different experimental conditions. Light-on is marked with *gray shading*. The difference between data points is 10 min. The short light phase jet lag larvae do not decrease their activity level directly when the lights go off. **b** Bar-graph representing the average activity at the last data point before the light went off (*black*) and the first two following data points after (*gray*). Significant differences of data points to the corresponding data point before are marked with *

**Fig. 6:**
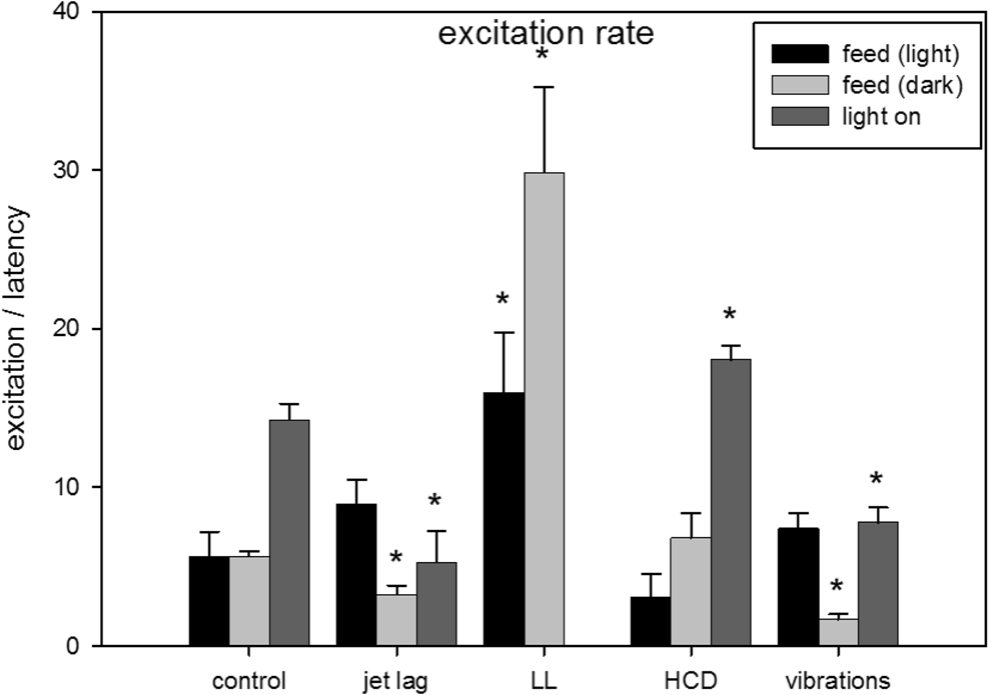
Excitation rate induced at lights-on or feeding during light or dark periods as mean over all events of an experimental period and over all individual experimental groups

Under control conditions (Table 1), a distinct light/dark pattern was observable with the resting phase of the larvae correlating with the dark cycle (Fig. 3a). The daily activity pattern recurred constantly during the whole observation period of 7 days. We calculated total activity (Fig. 4) and observed diurnal activity with very low activity levels (5 % of day activity) during the dark cycles (Fig. 4). The maximum activity of the light cycle was reached around 2 h after the light was switched on and 1 h after feeding and decreases steadily till the end of the light phase. When the light was switched off, the activity decreased rapidly till the resting level was reached (Fig. 5). During the dark phase, the activity level stayed rather constant with a short peak increase of activity when food was given (Fig. 3a). Responses to feeding were identical whether it was during the light or dark period (Fig. 6). To confirm that the response to feeding during the dark cycle was not only due to a mechanosensory stimulus, we added blank medium containing no Tetrahymena and no powdered food. Zebrafish larvae did not respond significantly to adding blank medium (data not shown). The strongest response in activity was observed when the light was switched on (Fig. 3a).

**Table 1:** Overview of the different treatment conditions (food, day/night cycle, vibration) applied in this study

Group	Treatment	Feeding
Control	14 h/10 h light/dark	*Tetrahymena* + baby food
Jet lag	Alternating prolonged and shortened days	*Tetrahymena* + baby food
LL	Continuous light	*Tetrahymena* + baby food
HCD	14 h/10 h light/dark	*Tetrahymena* + baby food + egg yolk
Vibrations	14 h/10 h light/dark, vibrations	*Tetrahymena* + baby food

### Altered rhythm of light/dark cycles

Under jet lag conditions (Table 1), zebrafish larvae still expressed a distinct day night activity pattern (Fig. 3b) but reduced total activity (Fig. 4) due to less activity during the light cycle compared to control animals (Fig. 4). Also excitation rate was decreased after lights-on and for feeding during the dark cycle (Fig. 6). Under control conditions, larvae reacted with a decrease in activity within 10 min after lights-off. By varying the timing of the light/dark switch during the jet lag protocol, the time it takes the larvae to respond to the lights-off was delayed (>10 min) (Fig. 5a). Control larvae showed a significant decrease in activity at the fist data point when the light went off, this was not the case for jet lag larvae (Fig. 5b). The time when the maximum activity during the light phase was reached did not correlate with the time the light went on but with the feeding time (Fig. 3b). When maximum activity was reached, the activity decreased steadily till the end of the light phase similar to control larvae (Fig. 3a/b).

When zebrafish larvae were exposed to LL conditions (Table 1), they still had coordinated resting cycles (Fig. 3c). Total activity was unaffected (Fig. 4) but activity in the control-dark-phase was increased and the activity at the control-light-phase was decreased. Some peaks of high maximal activity can be seen (Fig. 3c), these activity peaks were 20–40 % higher than the maximal activity in the control group (Fig. 3c). The larvae also showed higher excitation rates after feeding (Fig. 6). Peaks of activity correlate with timing of feeding (Fig. 3c).

### Altered caloric content of the food

HCD fed larvae (Table 1) showed continuously high activity levels during light cycles and significantly elevated activity levels during dark cycles (Fig. 3d). Thus, total activity was highest compared to all other monitored groups (Fig. 4). Also, excitation rate after lights-on was increased but not after feeding (Fig. 6). To get a closer look in the development of the activity changes, an experiment was performed were larvae were monitored from 5 dpf (1 day before feeding starts) till 10 dpf (Fig. 1b). Differences between treatments and time of development were analyzed using repeated measures ANOVA (*p*_adj_ < 0.05). Control larvae showed a significant increase in activity during the light phase from 5 dpf till 8 dpf, from 8dpf till 10 dpf no significant increase was observed (Fig. 3e). There was no significant difference between the activity of control and HCD larvae during the light phase till 10 dpf. During the dark phase, a slight significant shift in activity could be observed in control larvae. From 8 dpf HCD larvae showed a significant increase in activity during the dark phase compared to control larvae (Fig. 3e).

### Exposure to continuous vibrations

Exposure to sound/vibration (Table 1) lowered total activity levels (Fig. 4) particularly during the light cycle (Fig. 4) by lowering maximum activity (Fig. 3 F) but activity during the light period was still higher than during the dark. Furthermore, excitation rates after night feeding and lights-on were decreased (Fig. 6). During the sound/vibration experiment, a decrease in survival rate (∼50% during the 7-day sound exposure compared to 100%) was observed whereas no other treatment affected survival. Therefore, only the first 5 days of the experiment could be used for data analysis.

## Discussion

In higher vertebrates, active and sleeping periods are directly regulated by light and this is also the case in zebrafish (Burgess and Granato 2007). The pineal gland of zebrafish contains an intrinsic circadian clock that drives rhythmic synthesis of the hormone melatonin (Vatine et al. 2011). Melatonin, whose release is inhibited during light cycles, significantly and dose-dependently reduces zebrafish locomotor activity and increases arousal threshold (Zhdanova 2011). Thus, a distinct day/night activity pattern is expressed with diurnal activity. In all vertebrates locomotion is controlled by the nervous system, i.e., the brain and spinal cord, selecting appropriate motor microcircuits to create smooth and efficient movements and critical subcortical pathways are anatomically and functionally conserved throughout vertebrates (Grillner et al. 2013). Speed and direction of locomotion can be affected by neurodegenerative diseases and also by exposure to chemicals of neurotoxic potency but also visual, somatosensory and olfactory input can modulate the behavior of an organism. The aim of our study was to investigate how different external factors alter day/night activity.

### Circadian rhythms control activity levels

The circadian clock of an organism is regulated by endogenously driven cycles of roughly 24 h (circadian rhythms) and is needed to synchronize biochemical, physiological and behavioral processes. External cues like light, temperature, or food intake are used to drive the circadian clock.

When exposed to alternating light/dark cycles zebrafish larvae still display a distinct day night activity pattern. But varying the timing of the light/dark switch during the jet lag protocol, the time it took the larvae to reach their resting activity (latency) was delayed. This seems to indicate that the larvae’s response to lights is not only due to the change in illumination but it also depends on whether the larva anticipates the change or not.

When zebrafish larvae were exposed to continuous light they still showed rhythmic and synchronized resting periods. However, during the supposed resting periods, the activity levels were increased compared to control conditions. Even under continuous light, melatonin release shows a circadian pattern with reduced levels during the predicted night cycle (Danilenko et al. 2011). Exposure to continuous light also induced higher peaks in activity and increased excitability. Light is one of the strongest entrainment factors (zeitgeber) of circadian rhythms (Lopez-Olmeda et al. 2006) and directly stimulates the pineal gland. If the stimulus of changes in illumination is missing, like under continuous light conditions, circadian rhythms become free running or rather other rhythmic incidents like feeding could become the new zeitgeber. When larvae were exposed to continuous light for one week a new activity pattern was observable. Alterations in activity were mainly due to feeding but after three days, additional peaks occurred. Interestingly, these smaller peaks occurred around 9 h after the second feeding of the day. In our experiments, zebrafish larvae were fed twice a day with an interval of 9 and 15 h, respectively between feedings. Thus, feeding happened irregularly within 24 h. The newly observed activity peaks indicates that the zebrafish larvae anticipated the new zeitgeber (food) in a regular occurrence and aligned their activity levels. Within another study, we could show that LL fish show expression changes in circadian genes, which would confirm this hypothesis (Kopp et al. 2015).

### Food increases activity

At 5 dpf, when the swim bladder is fully inflated, zebrafish larvae start to effectively hunt for food (Strahle et al. 2012). Zebrafish larvae need their visual sense to track their pray (McElligott and O’Malley 2005) but during feeding zebrafish larvae also experience an olfactory stimulus. Olfactory inputs are processed in the forebrain and at 3–4 dpf zebrafish larvae already respond to amino acid odors with increased swimming activity (Lindsay and Vogt 2004) and are able to discriminate between some stimuli (Miyasaka et al. 2013). Adding blank medium during the dark cycle did not induce any response. This indicated that the response we saw after feeding during the dark cycle was only caused by the food itself. Under control conditions, excitation rates after feeding during light and dark cycles were equal. The same effect was seen in HCD fed zebrafish larvae. Based on these findings, we conclude that zebrafish respond to food irrespectively of what is fed.

Maximum activity was always reached during light phases after feeding, even when feeding occurred much later in the light phase (e.g., jet lag protocol). The strongest effect of food during light phases was seen under LL conditions, excitation rate after the first feeding was increased by a factor 2.8 while excitation rate after the second feeding, which equates the feeding during dark in control animals, was increased even by a factor of 5.3. This shows that besides the lights-on increase activity and feeding during light phases enhances activity.

Only under HCD conditions, total activity during light cycles was significantly increased compared to standard diet (1.3 fold) and also resting activity was increased by a factor of 3.5. This is interesting as it is normally assumed that HCD induces a decrease in activity. We could show that HCD fish also display altered expression of circadian and fat metabolism genes and an increase in adipocytes (Kopp et al. 2015). Alterations on behavior were visible after 2–3 days of HCD indicating that metabolic changes lead to the observed effects. The LL fish showed also a hyperactive phenotype and also displayed an increased number of adipocytes and did also alter circadian genes and genes of the fat metabolism (Kopp et al. 2015). The increased activity of LL larvae seemed to be caused by the lack of light/dark rhythms, the larvae become hyper excitable towards any external stimulus and do not show prober resting phases anymore. Since the diet of LL larvae is not different to control larvae, we assume that the increase in adipocytes is a compensatory effect due to the increased demand of energy. This could suggest that the behavioral hyperactive effects seen with HCD fish might be caused by similar metabolic changes as in LL fish and that the increase in adipocytes could also be a secondary effect due to increased energy demand instead of a direct effect of the high caloric diet. But to better understand the link between increased activity and fat metabolism in fish further research is needed.

### Stress reduces activity

In zebrafish, the hypothalamus-pituitary-interrenal axis regulates the adaptive response to stressors. Common stress response is acute and the adaptive changes only transient without causing damage to the organism. However, intense chronic stress over-stimulates the HPA axis, leading to a state of exhaustion, dysregulation of the stress system, and even death (Piato et al. 2011). When zebrafish larvae were stressed by continuous exposure to vibrations, total locomotor activity was significantly affected as the larvae became progressively less active during the light cycle and also excitation rate after lights-on was decreased. Interestingly activity during the dark cycle was not affected. Jet lag larvae induced a similar behavioral response on total and light phase dependent activity. Furthermore, in jet lag larvae and animals continuously exposed to vibrations, feeding during the dark cycle induced lower excitation rates than under control conditions. This indicated an increased arousal threshold which is characterized by lowered responsiveness to sensory or emotional stimuli (Pfaff et al. 2008). In the vibration-exposed larvae, the decrease in activity was followed by a reduced survival rate, indicating that the HPA axis might be over stimulated by the chronic stress. Interestingly the jet lag treatment did not reduce survival.

In conclusion, our study shows that altered timing of light dark cycles affected the larvae’s total activity and also excitation rates after lights on or feeding during the dark cycle. We could clearly demonstrate that zebrafish larvae align their locomotor activity to circadian entrainment factors. Regular stimuli induced different behavioral responses than unpredictable ones. Thus, anticipation of a certain stimuli due to habituation to certain rhythmicity induces responses of different excitation and latency. Changing the type of diet to HCD increased activity during light as well as dark cycles but it did not affect excitation rate when larvae were fed. So our results clearly demonstrated that continuous light affected the zebrafish larvae’s response to feeding and in turn the type of diet affected the response to changes in lightning. Exposure to continuous stress, i.e., vibrations extenuated zebrafish larvae’s response to light as well as feeding. Our results show that environmental factors as caloric content of food, light/dark cycles, and stress alter normal swimming behavior partially dramatically. Factors like light/dark cycles and vibrations altered behavior immediately whereas food content needed 2–3 days before alterations were visible, indicating that different molecular mechanisms might be involved. This results underline the high importance or rearing zebrafish under constant circadian rhythms (rhythmic changes in light/dark and feeding times) and setting comparable experimental time points as activity levels vary according to circadian time.
